# Should Malaria Treatment Be Guided by a Point of Care Rapid Test? A Threshold Approach to Malaria Management in Rural Burkina Faso

**DOI:** 10.1371/journal.pone.0058019

**Published:** 2013-03-05

**Authors:** Zeno Bisoffi, Halidou Tinto, Bienvenu Sodiomon Sirima, Federico Gobbi, Andrea Angheben, Dora Buonfrate, Jef Van den Ende

**Affiliations:** 1 Centre for Tropical Diseases, S. Cuore Hospital, Negrar, Verona, Italy; 2 Centre Muraz, Bobo Dioulasso, Burkina Faso; 3 Centre National de Recherche et de Formation sur le Paludisme, Ministry of Health, Ouagadougou, Burkina Faso; 4 Department of Clinical Sciences, Prince Leopold Institute of Tropical Medicine, Antwerp, Belgium; Johns Hopkins University, United States of America

## Abstract

**Background:**

In Burkina Faso, rapid diagnostic tests for malaria have been made recently available. Previously, malaria was managed clinically. This study aims at assessing which is the best management option of a febrile patient in a hyperendemic setting. Three alternatives are: treating presumptively, testing, or refraining from both test and treatment. The test threshold is the tradeoff between refraining and testing, the test-treatment threshold is the tradeoff between testing and treating. Only if the disease probability lies between the two should the test be used.

**Methods and Findings:**

Data for this analysis was obtained from previous studies on malaria rapid tests, involving 5220 patients. The thresholds were calculated, based on disease risk, treatment risk and cost, test accuracy and cost. The thresholds were then matched against the disease probability. For a febrile child under 5 in the dry season, the pre-test probability of clinical malaria (3.2%), was just above the test/treatment threshold. In the rainy season, that probability was 63%, largely above the test/treatment threshold. For febrile children >5 years and adults in the dry season, the probability was 1.7%, below the test threshold, while in the rainy season it was higher (25.1%), and situated between the two thresholds (3% and 60.9%), only if costs were not considered. If they were, neither testing nor treating with artemisinin combination treatments (ACT) would be recommended.

**Conclusions:**

A febrile child under 5 should be treated presumptively. In the dry season, the probability of clinical malaria in adults is so low, that neither testing nor treating with any regimen should be recommended. In the rainy season, if costs are considered, a febrile adult should not be tested, nor treated with ACT, but a possible alternative would be a presumptive treatment with amodiaquine plus sulfadoxine-pyrimethamine. If costs were not considered, testing would be recommended.

## Introduction

In health centers and dispensaries of many African countries, including Burkina Faso, malaria is the only disease for which a rapid diagnostic test (RDT) can be used in the field with immediate result. The diagnosis and management of all other clinical problems are entirely left to the clinical skills of trained nurses, as most of these peripheral health facilities have no doctor. Nurses should then follow clinical algorithms, that are designed to guide their decisions step by step, based on the presence/absence of clinical symptoms and signs, and more recently including malaria RDTs.

As far as the management of fever is concerned, local guidelines should follow what is now indicated by WHO for all malaria endemic countries: do the test (generally a RDT), treat for malaria if positive, refrain if negative [1]. Artemisinin combination treatments (ACT), that are highly effective, and also much more costly than previous regimens, are indicated as the drugs of choice in African countries where *P. falciparum* malaria prevails, including Burkina Faso. The test is indicated as mandatory in order to avoid drug overuse. A test is useful if the result is susceptible to change the decision that the clinical officer would make without test. This has not always been the case in previous studies on malaria RDT, showing that the negative RDT result did not prevent local health professionals from treating for malaria [2], [3]. Rather than passively adhere to suggested guidelines, health workers should be trained to deal with uncertainty on the basis of the best available evidence. This necessarily implies a clinical reasoning based on the threshold, a well known concept but which unfortunately has not yet duly influenced clinical practice [4], [5].

### Managing Uncertainty in Medicine: The Threshold Concept

The threshold notion is not new to clinical decision making. It was first introduced by Pauker and Kassirer with a memorable paper in the New England Journal of Medicine in 1975 [6]. Since then, the threshold has become a pivotal concept of evidence based medicine (EBM), and applications to many different fields of health care have been published[7]–[36]. Modern clinical decision-making could not prescind from the threshold analysis, whenever decisions need to be taken in absence of 100% certainty. In tropical medicine diagnostic facilities are usually limited. Nevertheless, the threshold concept is, unfortunately, largely foreign to this field.

### The Decision Threshold

**Figure 1:**
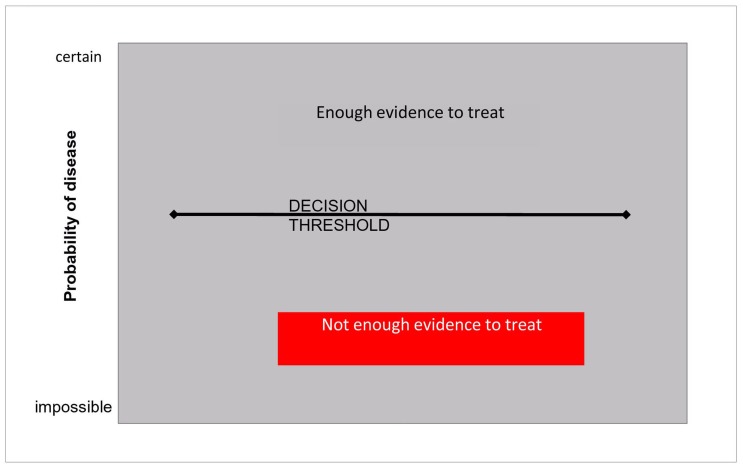
The (unique) treatment threshold (or decision threshold).

**Figure 2:**
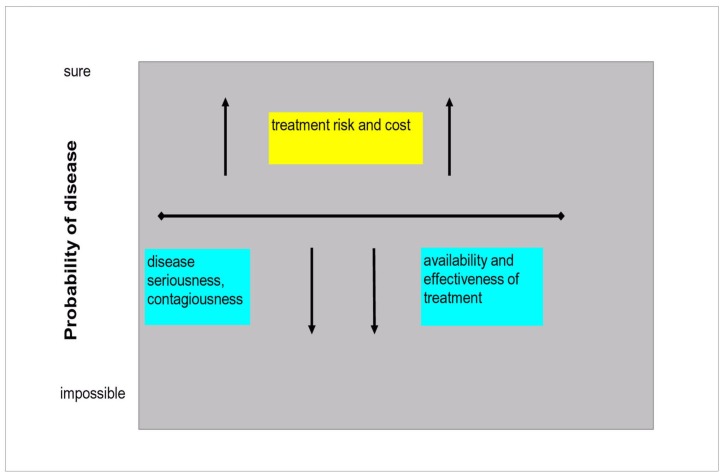
main factors influencing the decision threshold.

The probability for a patient to suffer from a given disease varies from 0% to 100%. The minimal probability required to decide whatever medical action (when all the available diagnostic arguments have been exhausted) is generally referred to as the treatment threshold [6]. A broader definition of “decision threshold” (Figure 1) is probably better, to comprise some decisions that do not concern treatment, such as: to communicate the diagnosis of an untreatable disease, or to refer to a higher level of care. If the decision concerns treating or not, which is usually the case, the threshold can be defined as a tradeoff between the consequences of refraining from the treatment when the disease is there and those of unnecessarily treating a patient who has not the disease (Figure 2).

### Factors Influencing the Decision Threshold

The disease and the treatment are the key factors affecting the decision threshold (Figure 2). More severe the disease is and lower the decision threshold will be, in order to minimize the number of “false negative” patients with the disease who would remain untreated. On the other hand, a treatment which is scarcely effective and/or very dangerous and/or very expensive and/or of limited availability will move the threshold upward. The consequences of a severe disease left untreated are generally much more dangerous than the undesired effects of the treatment; therefore the decision threshold for severe diseases is usually low [37]. Nevertheless, the treatment cost is a limiting factor. For this reason, the decision threshold for most diseases is higher in low income countries [4]. If a treatment is very expensive, it is not justified to treat many “false positives” with a high cost for the patient and/or for the community, depending on the payment system. An obvious example is the treatment of AIDS with protease inhibitors.

### When a “Last Test” is Available. Test Threshold and Test/Treatment Threshold

**Figure 3:**
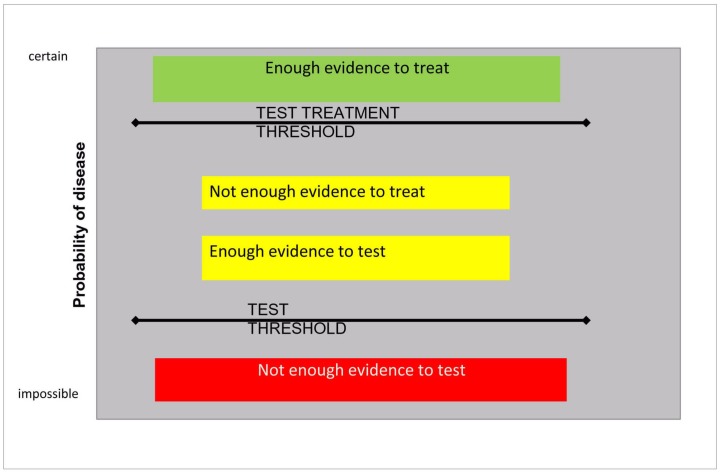
the test threshold and the test treatment threshold.

So far, a unique decision threshold has been considered: to treat or not. If we take malaria as an example, this is a potentially fatal disease, particularly for children under 5 years, and an effective treatment is available at a quite reasonable (though not negligible) cost. Therefore the decision threshold is low. In endemic countries, if no test is available, the simple presence of fever in a child justifies a presumptive treatment (meaning that the probability of disease is over the threshold). The introduction of a test, previously unavailable, such as malaria RDT, changes the logical framework. The test, contrarily to clinical arguments, has a cost, and should not be used if the result is irrelevant to the final decision. With the availability of the test, the decision is not simply to treat or not to treat. The clinician should first decide whether to test or not. It is logical to do the test, only if its result may change the ultimate decision. Therefore, the “final test” will act by “splitting” the decision threshold into two “new” thresholds [38](Figure 3):

1. **The test threshold**. It is the tradeoff between the decision to do nothing (or “exclude”) and the decision to do the test, and treat only if the test is positive.
2. **The test/treatment threshold**. It is the tradeoff between the decision to treat without test, and the decision to do the test and treat or not on the basis of its result.

Only when the disease probability lies between the two thresholds, should the clinician do the test (Figure 3).

### Factors Influencing the Test Threshold and the Test/Treatment Threshold

Obviously, the factors related to the disease and the treatment, that were discussed previously, will move the two thresholds in the same direction (upward and downward, respectively) as they do with the (unique) decision threshold. On the contrary, the factors related to the test move the two thresholds in opposite directions. In particular, the better is the sensitivity and specificity of a test, the wider the range of probabilities comprised between the two thresholds.

**Figure 4:**
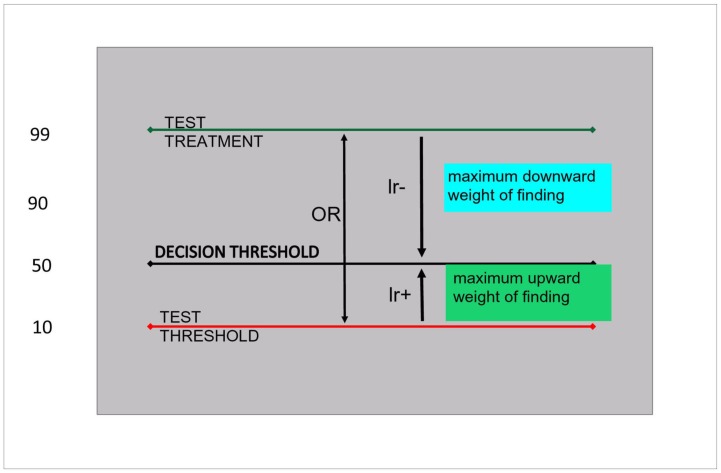
maximal theoretical effect of tests: odds ratio. Legend: OR: log_10_odds ratio; LR: log_10_likelihood ratio. Log_10_positive and negative likelihood summed make the log_10_odds ratio, shown on a log_10_odds scale. The width between test-treatment threshold and decision threshold is determined by the negative likelihood ratio of the test. Mutatis mutandis, the distance between the test treatment and the decision threshold are given by the positive likelihood ratio.

On a logarithmic probability scale it is easy to demonstrate that the maximum extension of this range (without considering costs) is mathematically represented by the test odds ratio, that expresses the whole accuracy of a test, resulting from the sensitivity and the specificity [39]. Figure 4 shows on a log10 probability scale the hypothetical effect of a test that would bring the probability down from 99% to 50%, if negative, or up from 10% to 50%, if positive. If the decision threshold is at 50%, the test-treatment threshold should then be located at 99%, since if it were higher, the negative test result would never bring the probability down to the decision threshold. Mutatis mutandis for the test threshold, this is located here not lower than at 10%, since this is the lowest probability from which the positive test result allows reaching the decision threshold. On this scale, the (log)odds ratio is the sum of the absolute values of (log)LR+ and the (log)LR-, as shown on the graph, and represents the maximal range of probabilities comprised between the two thresholds, as determined by the test accuracy [39].

Obviously, the test risk (if any) and cost will narrow this range, moving both thresholds toward the central, treatment (or decision) threshold.

### Case study. A Threshold Approach to Malaria Management in Rural Burkina Faso

What would be the decision threshold for malaria treatment in a health centre or dispensary in a malaria endemic area? Is it the same for an adult or a child? Intuitively, the answer to the first question is: a low threshold, considering that the risk of a missed treatment outweighs the negligible risk of an unnecessary treatment. As for the second question, malaria risk in a hyper endemic area is much higher for an infant or a child than for an adult, therefore the threshold is lower. With data obtained from previous studies in Burkina Faso [3], [40], the decision threshold for malaria management in adults and children will be first calculated (for the purpose of this study, we will call children all patients below 5 years, and adults those aged 5 years or more). Then, based on malaria RDT accuracy, we will also estimate the test and test/treatment threshold in the high and low transmission season. Thresholds will be first calculated based on health outcome only (mortality), in a second step including the cost of the test and of the treatment and the value attributed to a death averted. For adults, we will also estimate the thresholds using an alternative and less expensive antimalarial treatment, amodiaquine plus pyrimethamine-sulfadoxine.

## Methods

### Estimate of the Decision Threshold

The decision threshold *DT* is the level of probability at which the whole harm caused by the treatment equals the whole harm caused by the untreated disease. At *p* probability of disease, treating all will cause the same harm as treating nobody. If all are treated, all (100% or 1) will be exposed to the harm caused by treatment; if none is treated, the harm caused by the disease will concern *p* patients who have malaria.
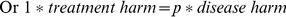
(1)


#### Harm expressed as mortality

In the simplest formulation, when only health outcome is considered, harm is represented by mortality caused either by the disease (*Dmort*) or by the treatment (*Tmort*). For *Dmort* the value of excess mortality will be used (obtained by subtracting from the total deaths due to the untreated disease those due to treatment failure). Then:
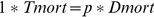
(2)


Then, by solving this simple equation to find the value of *p* that will correspond to DT:
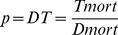
(3)


In a Cochrane review on different artemisinin combination treatments (ACT) for uncomplicated malaria, involving more than 20,000 patients overall [41], only very few deaths were recorded, and none was clearly attributable to the drug. We will use for our calculation a conservative estimate of 1 death per 10,000 treatments that is probably over rated. Malaria (untreated) excess mortality risk was estimated, using the findings from the previous study, at 3.7% for children and at 0.14% for adults [40]. The same estimates may reasonably apply to the alternative combination amodiaquine plus sulfadoxine-pyrimethamine, as this was found as effective as ACT in the study area [42]. As far as safety is concerned, amodiaquine is the partner drug of one of the most widely used ACTs, and the combination, as well as sulfadoxine-pyrimethamine alone, have been tested in several randomized controlled trials [42]–[45], while sulfadoxine-pyrimethamine has been extensively used as intermittent preventive treatment both in pregnancy and in infants, showing an excellent safety profile [46], [47].

#### Harm expressed as mortality plus cost

If costs are considered, then all variables must be attributed a monetary value, including life (or the value of a death averted). Treatment harm will add to the cost of treatment for all (*Tc*), the attributed value to a life (*Lc*) lost because of treatment toxicity, while the harm caused by the untreated disease will involve the attributed value to a life lost due to disease mortality.

By adding the new parameters, the following equation results:
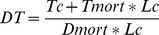
(4)


From the same source as above [40], an average treatment cost of 1 € for children and 2 € for adults were obtained for ACT (arthemeter-lumefantrine), while the value of a death averted (for both adults and children) was made vary between € 525 and 3150 (corresponding to YLL values of 25 and 150 US $, a proposed benchmark for a very cost-effective and a cost-effective health intervention, respectively) [2]. Costs are indicated in € as the local currency CFA has a fixed exchange rate with euro. The same conversion value with US $ as in the previous study [40] was maintained.

The thresholds were then calculated with both the lower and the upper values. The estimated average adult cost of an alternative treatment with amodiaquine plus sulfadoxine-pyrimethamine was of 0.14 € [48].

### Estimate of the Test and Test/Treatment Threshold

#### Estimate based on the test accuracy

With the data previously obtained on test accuracy for malaria-attributable fever and on the RDT cost [3], [40], the test threshold *t* and the test/treatment threshold *tT* were calculated using the formulas:
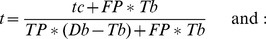
(5)

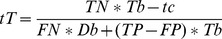
(6)Where: tc = test cost; FP = false positive rate (or 1-specificity); TP = true positive rate (or sensitivity); Tb = treatment burden ( = Tmort * Lc);

Db = disease burden ( = Dmort * Lc).

The derivation of both formulas for test and test/treatment thresholds are provided in Methods S1.

### Test and Treatment Decisions

The pre-test probability of malaria for a febrile patient of each age group and season, obtained from the same source, was then matched against the obtained test and test/treatment threshold. If the pre-test probability was below the test threshold, or above the test/treatment threshold, then the conclusion was that the test was not indicated.

### The Maximal Test Cost

The “maximal test cost” is the test cost that virtually eliminates the test range, making the two thresholds coincide with the decision threshold. Any test cost equal or above the maximal test cost makes the test a non viable option. The formula and its derivation is provided in Methods S1.

## Results

### Estimate of the Treatment or Decision Threshold

#### Harm expressed as mortality

If harm were considered only in terms of health outcome (mortality due to disease and treatment, respectively), applying Equation 3 to data on children, the value of DT would be:
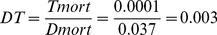



A calculated threshold in terms of mortality only, would be as low as 0.003, or 0.3%.

Applying the same equation to data on adults:
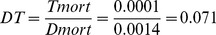



For adults, a calculated threshold in terms of mortality only, would be 0.071, or 7.1%.

#### Harm expressed as mortality plus cost

If costs are incorporated, using the highest limit of the range of value of a death averted for children (applying Equation 4) the decision threshold will be:
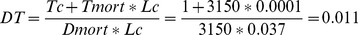



A threshold based on the higher value assigned to a death averted for malaria treatment in children is therefore 1.1%. If the lower value of a death averted is used, then the threshold would rise to 5.4% (calculations not shown).

For adults, at the higher value assigned to a death averted, the calculated threshold level would be 52.5% (calculation shown in Results S1). At the lower value, the whole cost of a treatment with ACT outweighs the benefits even at a 100% level of certainty.

Using for adults the alternative regimen of amodiaquine plus sulfadoxine-pyrimethamine, the threshold would be 0.103 (or 10.3%) at the higher value of a death averted (calculation shown in Results S1), and 0.262 (or 26.2%) at the lower value (calculation not shown). All the calculations hereafter will be based on the higher value.

### Estimate of the Test and Test/Treatment Threshold

#### Estimate of the test and test/treatment threshold without considering costs

For children, based on previously obtained data on test accuracy in the two seasons and on Equations 5 to 8 (calculations shown in Results S1), in the dry season the test threshold would be 0.08% and the test/treatment threshold 3.1%, while in the rainy season they would be 0.2% and 3.2%, respectively. For adults, the test and the test/treatment threshold would be 1.8% and 89.9% in the dry season, while in the rainy season 3% and 60.9%, respectively.

#### Test and test/treatment threshold including costs

For children in the dry season, the maximal test cost was 0.85 € while the real cost was 0.71 € (calculation shown in Results S1). The test and the test/treatment thresholds were 1.0% and 2.8%. In the rainy season, the maximal test cost was 0.44 € (largely below the real cost of 0.71 €), therefore the test option cannot be considered. For adults in the dry season the maximal test cost was 0.75 €, only slightly over the real cost; the test threshold was 50.6%, and the test/treatment threshold was 54.7%. In the rainy season, the maximal test cost for adults was 0.64 €, (below the real cost), therefore the test option was not viable. Using the alternative regimen for adults, the maximal test cost would be much lower than the real cost in both seasons (0.28 € and 0.24 € in the dry and rainy season, respectively).

#### Pre-test probability and the thresholds

The probability of malaria-attributable fever for children in the dry season was 3.2% [3], that is higher than the test/treatment threshold, whether costs are considered or not. In the rainy season the probability of malaria-attributable fever was 63.1%, that is much higher than the test/treatment threshold.

For adults in the dry season the pre-test probability of malaria-attributable fever in febrile patients was 1.7%, below the test threshold, both with and without considering costs. With the alternative regimen, the test was no more an option, and the disease probability was much lower than the decision threshold.

**Figure 5:**
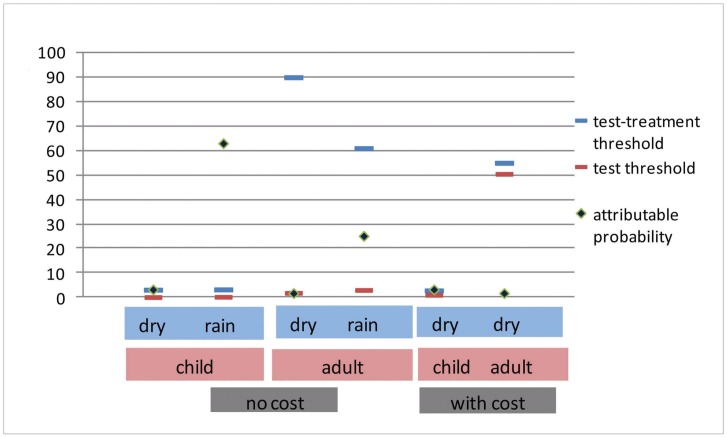
Summary of the relations between the pre-test probabilities and the thresholds. These are calculated both with and without considering costs (see text). When costs are considered, the test is no more an option in the rainy season (see article), while in the dry season the test field becomes very narrow as the two thresholds tend to coincide.

Finally, for adults in the rainy season the probability of malaria-attributable fever was 25.1%, that is, between the test and the test/treatment threshold without considering costs, while if costs were considered the test was not an option and the disease probability was lower than the decision threshold. With the alternative regimen of amodiaquine plus sulfadoxine-pyrimethamine, considering costs, the test was no more an option and the probability would be higher than the decision threshold. The relations between the pre-test probabilities and the thresholds are summarized in Figure 5.

In order to further illustrate the main results, four real case scenarios from the field studies are presented below. Clinical management will be first considered without a test, then with the availability of a RDT for malaria.

### Illustrative cases

#### Case 1

*At the end of May (end of the dry season) a 2-year-old boy is taken to a rural dispensary in the province of Banfora, Burkina Faso. He has got fever (38.5°C at the moment of consultation), the mother reports that he has been febrile for two days, has vomited twice and has a dry cough, no other significant clinical findings.*

Considering the local guidelines for presumptive management, without a test the nurse should treat for malaria any febrile case. In the dry season, the proportion of all fevers that is attributable to malaria is very low: only 3.2% [3]. The presence of vomiting slightly increases the probability of disease, but that of cough has the opposite effect (unpublished data). If the threshold of 1.1% is considered, then the nurse should treat, that is the right decision according to guidelines. The threshold based on mortality only, without costs, is even lower, then the decision would be the same. If a RDT is available, the probability of disease remains over the test/treatment threshold, considering costs or not : therefore, presumptive treatment remains the elective option.

#### Case 2

*At mid- October an 8-month-old girl is taken to the same dispensary with high fever (39°C) and vomiting. She breathes fast (52 respirations per minute). No cough. No clear pathologic finding at the chest auscultation.*

Again, the nurse should treat for malaria according to guidelines, if no test were available. In the high transmission season, malaria accounts for about two thirds of all fever cases [3]. Moreover, the presence of vomiting further increases the probability of malaria which is obviously much higher than the threshold (of 1.1% or 0.3% considering or not considering costs, respectively). The nurse should treat for malaria. With the availability of a RDT, WHO guidelines recommend testing, but the threshold-based analysis shows that the test should not be done, as a negative result would not change the decision to treat.

#### Case 3

*In April a 32-year-old local farmer consults for a 2-day fever, a slight headache and some “body pain”. He refers night sweats. The physical examination is normal. Temperature is 37.8°C.*

**Figure 6:**
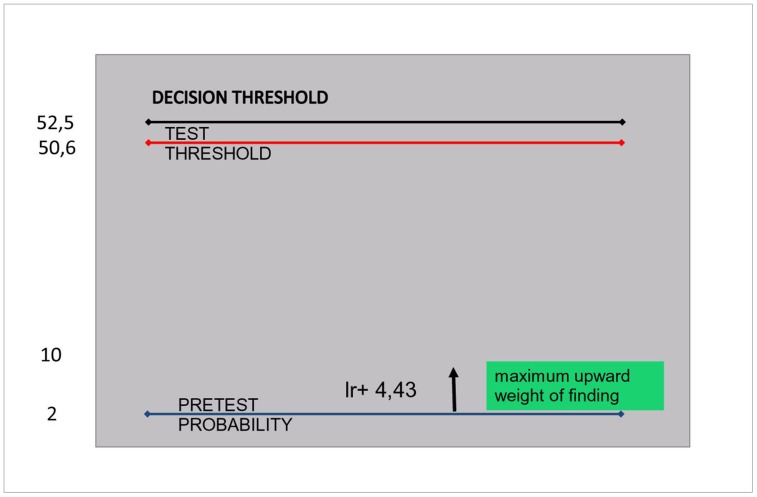
illustrative case 3: a febrile adult in the dry season. The pre test probability is at 2%, a positive RDT will never reach the decision threshold at 52.6 since its positive likelihood ratio is only 4.43.

Once again, the nurse should treat for malaria according to guidelines, in case no test is available. The probability of clinical malaria (dry season) is 1.7% only [3]. The treatment (or decision) threshold without costs is 7.1%, that based on the upper value attributed to a death averted is over 50% (while with the lower value an adult should never be treated with an ACT). According to the threshold the nurse should refrain from treatment. With an available RDT, the decision would not change in case of positive result, therefore the nurse should not use the test (Figure 6). Without considering costs, the conclusion would be the same. Even with the alternative regimen, the disease probability would remain below the decision threshold, and the test is not an option.

#### Case 4

*At the end of September a 19-year-old girl is taken to the same dispensary with fever (38.5°C) and no other major symptom nor clinical finding.*

Following guidelines the nurse should treat for malaria if a test is not available. For adults in the rainy season, malaria accounts for 25.1% of all fevers [3]. According to the threshold reasoning and without availability of RDT, the nurse should not treat with an ACT and investigate other possible (and more likely) causes of fever, since the decision threshold is situated at 52.5%, much higher than the probability of our patient, 25.1%. Only if costs were not taken into account the threshold (7.1%) would be lower than the disease probability. With the availability of a RDT, and without considering costs, the disease probability is situated between the test and the test/treatment threshold (3% and 60.9%, respectively), then the test should be used. Considering costs, the test is no more an option and the disease probability remains below the decision threshold, therefore the nurse should refrain from both test and treatment. With the alternative and cheaper regimen of amodiaquine plus sulfadoxine-pyrimethamine, the test would not be indicated either, while a presumptive treatment would, as the disease probability would be higher than the decision threshold (25.1% versus 10.2%).

## Discussion

### General Findings

As it has been previously shown, the generalized adoption of RDT for all ages in all malaria-endemic countries was not entirely based on evidence [49]. Nevertheless, the tests are now available (though with frequent shortages of supply) and local nurses should know when and how to use them in a rational way. If the threshold approach is used as a guide to individual clinical management, nurses in the study area should limit the use of RDT to a febrile adult in the rainy season. This is only true if all costs are entirely subsidized and therefore can be overlooked in the individual clinical context, while, if costs are considered, presumptive treatment becomes the correct choice, but only with the cheaper option. Children should be treated without test. Naturally, and independently on using or not the test and on the test result, nurses should carefully consider the clinical presentation for other potential causes: it is crucial to understand that reaching the threshold for a disease, in this case malaria, does not mean excluding other possible diseases. For example, in our Case 2 presented above, a fast respiratory rate would probably have indicated also a treatment with antibiotic for a possible pneumonia or sepsis.

In the dry season, and in both seasons if all costs are taken into account, adults should not be tested, nor treated with an ACT, if we consider fever management in the individual clinical context. This statement may appear particularly extravagant, but is clearly supported by the study results. This option (refraining from both test and treatment) was not considered in our previous paper on cost effectiveness of RDTs [40]: in that study the testing option was considered in alternative to the previous guidelines of presumptive treatment of all fevers. In a hyper endemic context such as the study area, malaria mortality risk is negligible for adults, and the treatment cost with ACT is high. Moreover, shortages of supply are frequent, and it seems reasonable to reserve the life-saving drug to children. A logical alternative for adults would be a presumptive treatment, in the high transmission season, with a cheaper drug combination such as amodiaquine plus sulfadoxine-pyrimethamine, that is still highly effective in the area [42]: testing should not be recommended, as the test cost would outweigh that of the drug. In the dry season, the probability of clinical malaria in adults is so low [3], that neither testing nor treating with any regimen should be recommended, unless fever does not subsides after treating for alternative and more likely causes. A comparison of WHO guidelines and the threshold-based analysis applied to our study setting is resumed in Table 1.

**Table 1:** A comparison of the general WHO guidelines with the possible recommendations for the study area, based on threshold analysis.

Management of a febrile patient	Child, dry season	Child, rainy season	Adult, dry season	Adult, rainy season
WHO guidelines	Test, treat for malaria if positive, consider other possible causes if negative	Id.	Id.	Id.
Threshold analysis, costs not considered	Treat for malaria without test, consider other possible causes	Id.	Refrain from both test and malaria treatment, consider other possible causes	Test, treat for malaria if positive, consider other possible causes regardless the result
Threshold analysis, costs considered	Treat for malaria without test, consider other possible causes	Id.	Refrain from both test and malaria treatment, consider other possible causes	Id., or treat for malaria with alternative regimen

x = diseased; 1-x = not diseased; Tc = Treatment cost; Tmort = mortality caused by the treatment; Lv = value of a death averted; Dmort = Disease mortality; t = test threshold; tT test/treatment threshold; tc = test cost; FP = false positive rate; TP = true positive rate; FN = false negative rate; TN = true negative rate; Tb = Treatment burden ( = Tc +Tmort * Lv); Db = Disease burden ( = Dmort * Lv).

Tc = Treatment cost; Tmort = mortality caused by the treatment; Lv = value of a death averted; Dmort = Disease mortality; t = test threshold; tT test/treatment threshold; tc = test cost; FP = false positive rate; TP = true positive rate; FN = false negative rate; TN = true negative rate; Tb = Treatment burden ( = Tc +Tmort * Lv); Db = Disease burden ( = Dmort * Lv).

Of course, in countries targeted for malaria elimination, malaria infection would deserve treatment in any case, in adults and children, and even if a fever may be due to other causes. The RDT should then be used for subjects without fever, too, who can be a reservoir of plasmodia. Our conclusions are by no means of general value, as they solely refer to a hyperendemic context where transmission intensity is still very high and malaria elimination is not yet a target.

### Comparison with Previous Studies

This is to our knowledge the first study using an explicit threshold approach to individual malaria management in the field. Several, previous cost-benefit and cost-effectiveness studies compared a test based versus presumptive management [40], [50]–[57]. This is the decisional level at the test/treatment threshold, but in general, the options considered did not include that of refraining from both the test and the treatment, which concerns the decisional level at the test threshold. Both decisional levels are equally important and should be considered in the analysis of any strategy.

### Weaknesses and Limitations

A number of limitations to this study should be acknowledged. Some of the parameters used for the threshold calculation are based on assumptions and/or expert opinion and might be questioned, such as the mortality attributed to ACT that might well be overestimated. Moreover, assigning an explicit monetary value to a death averted is obviously distasteful, but this is what is implicitly done in practice, as resources are not unlimited. Even attributing an unlimited value to human life and not considering the test and treatment costs, however, only for adults in the rainy season would the main conclusions change, with testing becoming the preferred option. Some of the study estimates are questionable, such as malaria mortality of adults and children, that are based, though, on primary data obtained in the field. Moreover other values are not considered, as they are very difficult to estimate: among them, morbidity and the consequent disability and loss of working days. These limitations, though, concern the data and not the methodological, threshold-based approach, that we believe is rigorous and robust in itself.

### Possible Impact

This study questions the generalized use of RDTs in all endemic settings, which is a concern shared by others [49], [58]. From a practical point of view, it is not easy to adopt a different policy by season and/or by age group, as the intensity of malaria transmission varies over time and it may be impossible to establish definite periods for using and not using the test. It may be equally difficult in real life refraining from a test when this is available, or reserve its use to a given age group only. For children, the more logical solution in the study setting would be returning to a presumptive malaria management all-year-long, at least until malaria incidence declines to a level that justifies a test-based policy.

For adults, the study results question the issue of ACT use in a highly endemic setting that is still far from being targeted for malaria elimination. Also in view of the growing concern about the possible appearance in Africa of *P. falciparum* strains with mutations linked to artemisinin resistance [59], a discussion about a possible, more focused use of ACT would be welcome. More in general, an evidence-based approach to clinical decision-making in tropical medicine would certainly take advantage from the threshold-based reasoning.

## Supporting Information

Methods S1 **variables.** x = diseased; 1-x = not diseased; Tc = Treatment cost; Tmort = mortality caused by the treatment; Lv = value of a death averted; Dmort = Disease mortality; t = test threshold; tT test/treatment threshold; tc = test cost; FP = false positive rate; TP = true positive rate; FN = false negative rate; TN = true negative rate; Tb = Treatment burden ( = Tc +Tmort * Lv); Db = Disease burden ( = Dmort * Lv).
(DOC)

Results S1 **variables**
Tc = Treatment cost; Tmort = mortality caused by the treatment; Lv = value of a death averted; Dmort = Disease mortality; t = test threshold; tT test/treatment threshold; tc = test cost; FP = false positive rate; TP = true positive rate; FN = false negative rate; TN = true negative rate; Tb = Treatment burden ( = Tc +Tmort * Lv); Db = Disease burden ( = Dmort * Lv).
(DOC)
