# Supplementary material for: Should Malaria Treatment Be Guided by a Point of Care Rapid Test? A Threshold Approach to Malaria Management in Rural Burkina Faso
Source: PLoS One. 2013 Mar 5;8(3):e58019. doi: 10.1371/journal.pone.0058019 (PMC3589446; doi:10.1371/journal.pone.0058019)
Supplement: Methods S1 — variables. x = diseased; 1-x = not diseased; Tc = Treatment cost; Tmort = mortality caused by the treatment; Lv = value of a death averted; Dmort = Disease mortality; t = test threshold; tT test/treatment threshold; tc = test cost; FP = false positive rate; TP = true positive rate; FN = false negative rate; TN = true negative rate; Tb = Treatment burden ( = Tc +Tmort * Lv); Db = Disease burden ( = Dmort * Lv). (DOC) [file pone.0058019.s001.doc]

**Methods S1**

**Derivation of the formulas for the test threshold, the test/treatment threshold and the maximal test cost[[1]](#footnote-2)**

### TEST THRESHOLD

**Disutilities of testing** = tc + x*Db*FN + x*Tb*TP + (1-x)*FP*Tb

**Disutilities of refraining** = x*Db

x*Db = tc + x*Db*FN + x*Tb*TP + (1-x)*FP*Tb

x*Db - x*Db*FN - x*Tb*TP - FP*Tb + x*FP*Tb = tc

x*TP*Db - x*Tb*TP + x*FP*Tb = tc + FP*Tb

$x=t=\frac{\text{tc}+\text{FP*Tb}}{TP*Db-TP*Tb+FP*Tb}=\frac{tc+FP*Tb}{TP*(Db-Tb)+FP*Tb}$**(Equation 1)**

### TEST/TREATMENT THRESHOLD

**Disutilities of testing** = tc + x*FN* Db + (1-x)* FP * Tb + x*TP*Tb

**Disutilities of treating** = Tb

tc + x*FN* Db + (1-x)* FP * Tb + x*TP*Tb = Tb

Tb – tc = x*FN* Db + (1-x)* FP * Tb + x*TP*Tb

Tb – tc = x*FN* Db + FP * Tb – x* FP * Tb + x*TP*Tb

Tb - FP * Tb – tc = x*FN* Db – x* FP * Tb + x*TP*Tb

TN*Tb – tc = x* (FN*Db - FP * Tb + TP*Tb)

$x=tT=\frac{\text{ TN*Tb - tc}}{FN*Db+TP*Tb-FP*Tb}=\frac{TN*Tb-tc}{FN*Db+(TP-FP)*Tb}$**(Equation 2)**

###

### MAXIMAL TEST COST (Mtc)

It is the test cost (or cost plus risk) which (for a given Decision Threshold DT and a given test accuracy) reduces the test field to 0 (option of testing equals options of not testing). If tc = Mtc, then all options will be equivalent (there will be no more test field). If tc > Mtc, then the calculations of t and tT will not make sense, as the test will never be an option.

The first “tradeoff” will be between testing **all** (at DT probability of disease) and treat the positives, **or** not testing anybody and treat **all**.

**Decision threshold** (without test): $DT=\frac{\text{ Tb}}{Db}$**(Equation 3)[[2]](#footnote-3)**

**Disutilities of testing** = test cost + Dis burden for FN + Treat burden for FP + Treat burden for TP

**Disutilities of treating** = test cost + Dis burden for FN + Treat burden for FP + Tb for TP

Mtc + Db * DT * FN + Tb * DT* (1-FN) +Tb * (1-DT) * FP = Tb

For equation 3, I can replace Db*Dt with Tb, then:

Mtc + Tb * FN + Tb * DT* (1-FN) +Tb * (1-DT) * FP = Tb

Mtc = Tb - Tb * FN - Tb * DT* (1-FN) - Tb * (1-DT) * FP

Mtc = Tb (1 – FN) - Tb * DT* (1-FN) - Tb * (1-DT) * FP

Mtc = Tb (1 – FN)*(1 – DT) - Tb * (1-DT) * FP

Mtc = Tb * (1-DT)*(1-FN-FP) = Tb * (1-DT)*(1-(1-Sens)-(1-Sp)) = Tb * (1-DT)*( Sens+Sp -1)

**Mtc = Tb * (1-DT)*( Sens+Sp -1) (Equation 4)**

The second “tradeoff” would be between testing 100% (at DT probability of disease) and treat the positive **or** doing nothing (with the Disease burden for DT)

Mtc + Db * DT * FN + Tb * DT* (1-FN) +Tb * (1-DT) * FP = Db * DT

But, based on Equation 1, we can substitute the second part of the equation with TrC, obtaining the same Equation 4).

1. ^^ Formulas are originally derived and differ from Pauker and Kassirer’s cited in the reference list [↑](#footnote-ref-2)
2. ^^ The derivation of the formula for DT is presented in the main article [↑](#footnote-ref-3)
