# Supplementary material for: Should Malaria Treatment Be Guided by a Point of Care Rapid Test? A Threshold Approach to Malaria Management in Rural Burkina Faso
Source: PLoS One. 2013 Mar 5;8(3):e58019. doi: 10.1371/journal.pone.0058019 (PMC3589446; doi:10.1371/journal.pone.0058019)
Supplement: Results S1 — variables Tc = Treatment cost; Tmort = mortality caused by the treatment; Lv = value of a death averted; Dmort = Disease mortality; t = test threshold; tT test/treatment threshold; tc = test cost; FP = false positive rate; TP = true positive rate; FN = false negative rate; TN = true negative rate; Tb = Treatment burden ( = Tc +Tmort * Lv); Db = Disease burden ( = Dmort * Lv). (DOC) [file pone.0058019.s002.doc]

**Results S1 - Calculations**

# Calculation of the treatment threshold or decision threshold (DT) for adults, including costs

Calculations based on Equation 4 of the article.

$$DT=\frac{Tc+Tmort*Lc}{Dmort*Lc}=\frac{2+3150*0.0001}{3150*0.0014}=0.52$$

The decision threshold for adults using the alternative regimen (amodiaquine plus sulfadoxine-pyrimethamine) will be obtained by substituting the average treatment cost of ACT with that of the alternative regimen, that is, 0.14 €.

$$DT=\frac{0.14+3150*0.0001}{3150*0.0014}=0.103$$

# Calculation of the test and test treatment threshold for children and adults in the two seasons

Calculations based on Equation 5 and Equation 6 of the article. For derivation, see Supplement 1

$t=\frac{tc+FP*Tb}{TP*(Db-Tb)+FP*Tb}$ (Equation 5) and:

$tT=\frac{TN*Tb-tc}{FN*Db+(TP-FP)*Tb}$ (Equation 6)

**1) Test threshold**

1. Children, dry season

$t=\frac{0.71+0.29\text{*(1}+\text{0.0001*3150)}}{0.94*0.037*3150-(0.94+0.29)*(1+0.0001*3150)}=0.010$ or 1.0%

If costs were not considered, then the result would be: t = 0.0008 or 0.08%

1. Children, rainy season

The calculations only make sense without considering costs:

$t=\frac{0.63\text{*0.0001}}{0.97*(0.037-0.0001)+0.63*0.0001}=0.002$ or 0.2%

If costs were considered, the calculation would give an aberrant result, as the real test cost is higher than the Mtc (see Supplement 1)

1. Adults, dry season

$t=\frac{0.71+0.217\text{*(2}+\text{0.0001*3150)}}{0.903*0.0014*3150-(0.903+0.217)*(2+0.0001*3150)}=0.506$ or 50.6%

If costs were not considered, then the result would be: t = 0.018 or 1.8%

1. Adults, rainy season

The calculations only make sense without considering costs:

$t=\frac{0.392\text{*0.0001}}{0.97*0.0014-(0.97+0.392)*0.0001}=0.030$ or 3%

If the test cost was considered, the calculation would give an aberrant result, as the real test cost is higher than the Mtc (see below)

**2) Test/treatment threshold**

1. Children, dry season

$tT=\frac{\text{0.71*(1}+\text{0.0001*3150)-0.71}}{0.06*0.037*3150+(0.94-0.29)*(1+0.0001*3150)}=0.028$ or 2.8%

If costs were not considered, then the result would be: t = 0.031 or 3.1%

1. Children, rainy season

The calculations only make sense without considering costs:

$tT=\frac{\text{0.37*0.0001}}{0.03*0.037+(0.97-0.63)*0.0001}=0.032$ or 3.2%

If costs were considered, the calculation would give an aberrant result, as the real test cost is higher than the MTC (see Supplement 1)

1. Adults, dry season

$tT=\frac{\text{0.783*(2}+\text{0.0001*3150)-0.71}}{0.097*0.0014*3150+(0.903-0.217)*(1+0.0001*3150)}=0.547$ or 54.7%

If costs were not considered, then the result would be: t = 0.383 or 38.3%

1. Adults, rainy season

The calculations only make sense without considering costs:

$t=\frac{0.608\text{*0.0001}}{0.003*0.0014+(0.97-0.392)*0.0001}=0.609$ or 60.9%

If costs were considered, the calculation would give an aberrant result, as the real test cost is higher than the Mtc (see below)

The test and test/treatment threshold for adults using the alternative regimen (amodiaquine plus sulfadoxine-pyrimethamine) will be obtained by substituting the average treatment cost of ACT with that of the alternative regimen, that is, 0.14 €. In this case, the real test cost is higher than the Mtc (see below), then testing would never be an option.

# Calculation of the maximal test cost (Mtc) for children and adults in the two seasons

Calculations based on Equation 4 of Supporting Information S1

1. Children, dry season

Mtc = $\text{(1}+\text{0.0001*3150)}$*(1 – 0.0113)*(0.94+0.71-1)= 0.85

1. Children, rainy season

Mtc = $\text{(1}+\text{0.0001*3150)}$*(1 – 0.0113)*(0.97+0.37-1)= 0.44

1. Adults, dry season

Mtc = $\text{(2}+\text{0.0001*3150)}$*(1 – 0.525)*(0.94+0.71-1) = 0.75

1. Adults, rainy season

Mtc = $\text{(2}+\text{0.0001*3150)}$*(1 - 0.525)*(0.97+0.61-1) = 0.64

The Mtc for adults using the alternative regimen (amodiaquine plus sulfadoxine-pyrimethamine) will be obtained by substituting the average treatment cost of ACT with that of the alternative regimen, that is, 0.14 €, resulting:

Dry season: Mtc = 0.28

Rainy season: Mtc = 0.24
